# Functional CdS-Au Nanocomposite for Efficient Photocatalytic, Photosensitizing, and Two-Photon Applications

**DOI:** 10.3390/nano10040715

**Published:** 2020-04-10

**Authors:** Katarzyna C. Nawrot, Dominika Wawrzyńczyk, Oleksii Bezkrovnyi, Leszek Kępiński, Bartłomiej Cichy, Marek Samoć, Marcin Nyk

**Affiliations:** 1 Advanced Materials Engineering and Modelling Group, Faculty of Chemistry, Wroclaw University of Science and Technology, Wybrzeze Wyspianskiego 27, 50-370 Wroclaw, Poland; katarzyna.nawrot@pwr.edu.pl (K.C.N.); dominika.wawrzynczyk@pwr.edu.pl (D.W.); marek.samoc@pwr.edu.pl (M.S.); 2 W. Trzebiatowski Institute of Low Temperature and Structure Research Polish Academy of Sciences, Okólna 2, 50-422 Wroclaw, Poland; o.bezkrovnyi@int.pan.wroc.pl (O.B.); l.kepinski@int.pan.wroc.pl (L.K.); b.cichy@int.pan.wroc.pl (B.C.)

**Keywords:** hybrid nanoparticle, quantum dots, gold nanoparticles, photocatalysis, reactive oxygen species generation, two-photon absorption

## Abstract

We demonstrate a low-temperature synthesis of hydrophilic, penicillamine-stabilized hybrid CdS-Au nanoparticles (NPs) utilizing different Au concentrations. The obtained hybrid nanomaterials exhibit photoluminescence quenching and emission lifetime reduction in comparison with their raw semiconductor CdS NPs counterparts. An increase of concentration of Au present at the surface of CdS leads to lower photoluminescence intensity and faster emission decays, suggesting more efficient charge separation when larger Au domains are present. For photocatalysis studies, we performed methylene blue (MB) absorption measurements under irradiation in the presence of CdS-Au NPs. After 1 h of light exposure, we observed the absorbance decrease to about 35% and 10% of the initial value for the CdS-5Au and CdS-7.5Au (the hybrid NPs obtained in a presence of 5.0 and 7.5 mM Au), respectively, which indicates MB reduction caused by electrons effectively separated from holes on metal surface. In further similar photocatalysis experiments, we measured bovine serum albumin (BSA) integrated photoluminescence intensity quenching in the presence of CdS-Au NPs, with a 50% decrease being obtained for CdS-2.5Au NPs and CdS-5Au NPs, with a faster response rate detected for the system prepared with a higher Au concentration. The results suggest hole-driven reactive oxygen species (ROS) production, causing BSA degeneration. Finally, we performed two-photon excited emission (TPEE) measurements for CdS-5Au NPs, obtaining their two-photon absorption (TPA) cross-section values up to 15.8 × 10^3^ GM (Goeppert-Mayer units). We conclude that the obtained water-soluble CdS-Au NPs exhibit potential triple functionalities as photocatalysts for reduction and oxidation reactions as well as materials for two-photon absorption applications, so that they may be considered as future theranostics.

## 1. Introduction

A popular trend of novel material engineering is to design more advanced materials, especially nanomaterials, which display several functionalities at one time. Those materials are considered useful in such fields as, among many others, medicine, energy generation, conversion, and storage. For example, medical multifunctional materials may be designed to enable simultaneous diagnosis and therapy (theranostics) [1]. In the case of energy, multifunctionality may be used for so-called ‘smart windows’, which possess not only energy saving, but also storing ability [2], while efficient energy generation may be achieved by combining ability to obtain sufficient voltage output, conversion efficiency, and stability [2]. Moreover, the wide possibilities of tuning materials properties by modifying their structure or composition allow one to combine also less trivial characteristics for future applications.

Among various functionalities that may be engineered into materials, catalytic properties merit attention. Catalysts are often necessary and even irreplaceable to carry out certain chemical reactions. As they are not reactants, they are not consumed and processed to products during reaction. They are often used in extremely small concentrations in comparison with those of the reactants. Without catalysts, the lion’s share of industrial, laboratory, and biological chemical reactions would be inefficient or even impossible to perform, at least at ambient temperature. Nowadays, photocatalysts gain particular interest as they enable conversion of, for example, solar into chemical energy, allowing chemical reactions to occur. A very important branch of photo-driven catalytic reactions is those using nanoparticles (NPs) as a controlling factor in processes such as wastewater treatment [3,4], CO_2_ reduction [5,6], or hydrogen generation [7,8].

Hybrid nanosystems, such as ZnO nanoparticles with Au domains [9,10], Au tipped CdSe nanorods [11], Pt-decorated CdS nanorods [12], CdSe nanoplatelets with Pd domains [13], Cu_2_O NPs with Au core [14], and even more complex systems like TiO_2_ nanowires enriched with Au NPs and deposited Cu_7_S_4_ [15], have been recently proposed as photocatalysts and solar energy converters. A very interesting approach was proposed by Yu et al. [16], who synthesized Ag/AgCl/TiO_2_ nanotube arrays and demonstrated their photocatalytic activity, taking visible light-driven methyl orange degradation as an example. Another inspiring work was done by Vaiano et al. [17] as they prepared ZnO NPs modified with Ag, showing their ability to support photocatalytic degradation of caffeine. Moreover, Han et al. [18] proposed a simple synthesis of highly active spherical CdS-Au NPs for environmental pollution photocatalytic reduction. Those hybrid nanosystems were composed of noble metals nanostructures grown on semiconductor NPs surface. The close vicinity of semiconducting quantum dots (QDs) and metallic NPs has consequences for their optical properties, especially those of the semiconducting part, which are different in comparison with their individual components. When a semiconductor is excited with light of sufficient energy, electrons from its valence band are promoted to the conduction band. However, the radiative recombination process is very quick (usually on the scale of tens of nanoseconds), so positive or negative charge cannot be harnessed for desired catalytic processes separately. In the case of hybrid nanosystems, photoexcited electrons are transferred to the metal part, preventing the electron–hole emissive recombination, and thus allowing for electron- or hole-dependent processes to occur. Therefore, semiconductor–metal heteroparticles may serve as efficient photocatalysts supporting both reduction and oxidation reactions.

Redox properties of nanosystems may be investigated using several methods. For reduction ability studies, absorbance of organic dyes, which do not absorb light in their reduced forms, is measured when they are mixed with NPs of interests and exposed to light. Rhodamine B [19] or methylene blue (MB) [11] are common choices for such experiments. Another popular method is based on measurements of hydrogen generation resulting from water splitting induced by light in the presence of selected nanosystems [20]. In order to separate holes from electrons even more efficiently, so-called hole scavengers, for example, lower alcohols, are added to the mixture. Oxidation ability of nanosystems suspended in water can also manifest itself by reactive oxygen species (ROS) generation, which, on the other hand, is of great potential for photodynamic-based cancer treatments. They may be detected using electron spin resonance spectroscopy [9] or indirectly by photoluminescence measurements of ROS-sensitive compounds mixed with the studied NPs and exposed to light. ROS generation not only serves as a proof of oxidation reaction occurrence, but also is beneficial itself, as it is able to induce cells death, thus opening another range of hybrid metal–semiconductor nanosystems applications; that is, as antimicrobial agents or for mutated cell death in targeted therapies.

An interesting group of photoactive materials is those that exhibit nonlinear optical (NLO) properties, especially those showing large multiphoton absorption cross sections. They are widely considered as optical agents for applications requiring longer excitation wavelengths such as in medical diagnosis, for instance, photodynamic two-photon cancer therapy or two-photon bioimaging, where excitation in the range of biological transmission window (650–900 nm) is preferred [21,22]. In fact, semiconductor nanoparticles are being widely characterized with respect to their NLO characteristics, as they possess significant advantages for the above-mentioned applications such as stability upon high-energy light exposure (no bleaching) and high luminescence quantum yields [23,24]. They have been reported to display large two-photon absorption (TPA) cross sections with maxima of up to 10^3^ GM for PbS [25] and InP@ZnS [24] QDs; up to 10^4^ GM order of magnitude for CdS [23,26,27], CdSe [28,29], and PbSe [25] QDs; or even larger for 1D and 2D nanoparticles, depending on the size, shape, medium, and characterization method.

For many applications, not only the simultaneous absorption of two photons, but also the ability of a material to emit the absorbed energy in the form of radiation is crucial. Therefore, not only the TPA cross section itself, but rather the two-photon brightness parameter, which is the product of the TPA cross section and the quantum yield (QY), characterizes the usefulness of a material in such applications. Owing to their high QYs, semiconductor NPs exhibit significant two-photon brightness, however, some types of their functionalization may reduce this essential parameter. For instance, the two-photon excited fluorescence may be bleached when metal domains are embedded in the structure of semiconductor nanomaterial because they can take over electrons from excitons and prevent recombination of electron-hole pairs. In this study, we present a low-temperature synthesis, photocatalytic ability and optical characterization, including NLO properties, of water-soluble penicillamine-stabilized CdS NPs with gold nanostructures attached to the surface. As they are hydrophilic, they may be directly applied as photocatalysts of reactions performed in water, and no post-synthetic surface treatment for aqueous phase transfer is necessary. After assessing the influence of the amount of Au seeded at the CdS NPs’ surface on their optical properties, we further show their ability to catalyze reduction reactions at room temperature, taking as an example MB photocatalytic reduction monitored by measurements of MB absorption against the irradiation time. Moreover, we compare their photocatalytic effectiveness depending on Au content. By detecting bovine serum albumin (BSA) photoluminescence quenching in the presence of CdS-Au hybrid nanosystems, we investigate their ability to generate ROS directly from surrounding water. We also characterized NLO properties of the hybrid material and report on optimal concentration of Au NPs embedded in CdS NPs, which introduces photocatalytic properties for both reduction and oxidation reactions, preserving their TPA and brightness abilities.

## 2. Materials and Methods

D-penicillamine (98%), L-penicillamine (99%), anhydrous sodium hydroxide (≥98%, pellets), cadmium chloride hydrate (CdCl_2_, 98%), and thioacetamide (≥99%) were purchased from Sigma Aldrich (Warsaw, Poland) and used for CdS NPs synthesis. Gold (III) chloride hydrate (HAuCl_4_, 99.995%) for gold nanostructures synthesis, as well as sodium borohydride (NaBH_4_, ≥96%) for gold ions reduction, were also purchased from Sigma Aldrich. Methylene blue (MB, POCH, Poland) and ethyl alcohol (96%, POCH, Poland) were used for photocatalytic activity measurements. ROS generation ability was investigated using bovine serum albumin (BSA, 67 kDa, Sigma Aldrich).

### 2.1. Synthesis

#### 2.1.1. CdS NPs

The synthesis of CdS NPs was carried out based on the method described by Moloney et al. [30]. First, 10 mL of 0.01 M racemic penicillamine solution and 40 mL of distilled water were mixed in a 250 mL three-neck flask. Then, 2 M NaOH solution was poured dropwise in order to set pH of the mixture to about 11–12 for the sake of disulphide formation prevention. Next, 8 mL of 0.01 M CdCl_2_ and 2 mL of 0.01 M thioacetamide were added to the alkaline solution. The mixture was heated up under nitrogen and kept at 100 °C for 2 h. The solution was then cooled to room temperature and kept overnight in the flask wrapped with aluminium foil. The material was concentrated by vacuum rotary evaporator, gently centrifuged for 2 min at 1800 rpm, and decanted clear yellow solution was separated from possible aggregates. Finally, the solution was filtered using 300,000 MWCO (molecular weight cut-off) centrifugation filters giving 4 mL of penicilline-stabilized CdS NPs in aqueous solution.

#### 2.1.2. CdS-Au Hybrid Nanosystems

The CdS-Au hybrid nanosystems were obtained based on gold ions reduction at the surface of CdS NPs. First, 1 mL of as-synthesized CdS NPs was quickly added to 1 mL of 1 mM HAuCl_4_ aqueous solution with continuous stirring. The colour of the solution changed to light brown and the mixture was kept stirred for at least 1 h. The process was repeated using 2.5 mM, 5 mM, and 7.5 mM concentration of HAuCl_4_ in order to investigate the impact of quantity of Au present at the CdS NPs surface on their optical properties, photocatalytic, and ROS generation ability of the hybrid systems; the resulting systems will hereinafter be called CdS, CdS-2.5Au, CdS-5Au, and CdS-7.5Au, respectively.

#### 2.1.3. Au Nanostructures

The free Au nanostructures for comparison with the CdS-Au hybrid nanosystems were also synthesized. Briefly, 1 mL of 1 mM HAuCl_4_ was mixed with Rac-penicillamine solution (750 μL, concentration corresponding with the one used for the CdS NPs synthesis). Then, 36.5 mg of NaBH_4_ was dissolved in 5 mL of cold water, and 250 μL of the solution was quickly introduced into the mixture of gold ions and ligands. The mixture was kept stirred for at least 1 h. The process was repeated using 2.5 mM, 5 mM, and 7.5 mM concentration of HAuCl_4_.

### 2.2. Characterization Methods

#### 2.2.1. Morphology and Spectroscopy Characterization

The morphology of the synthesized CdS-Au NPs was examined with transmission electron microscopy (TEM) using two electron microscopes: a FEI Tecnai G^2^ 20 X-TWIN microscope with EDX and a PhilipsCM-20 SuperTwin instrument operating at 160 kV. The diluted samples for TEM measurements were ultrasonicated, and a droplet of the suspension was deposited on a TEM dedicated copper grid coated with carbon film.

Initial optical characterization included absorbance and luminescence spectra measurements of the obtained CdS-Au hybrid systems, as well as as-synthesized penicillamine-stabilized CdS NPs along with time-resolved luminescence decay traces. Absorbance measurements were performed with a JASCO V670 spectrophotometer. Luminescence spectra were obtained using a Hitachi F-4500 spectrofluorometer, after excitation at λ = 375 nm. Luminescence lifetimes were measured with a self-constructed time-correlated single-photon counting (TCSPC) Becker & Hickl system (Berlin, Germany), constructed from a TCSPC Module (SPC-130-EM) and a hybrid PMT detector (HPM-100-06) with a detector control card (DCC 100) mounted onto a Princeton Instruments spectrograph (ActonSpectraPro-2300i) under excitation with a picosecond 375 nm laser diode (BDL-375-SMC). The luminescence lifetime values were calculated based on the exponential decay model, with the use of the dedicated Becker & Hickl SPCImage software.

#### 2.2.2. Photocatalytic Activity Measurements

Our main goal was to investigate the influence of the Au nanostructures at the CdS NPs’ surface on their optical properties, and simultaneously, the photocatalytic activity of those hybrid nanosystems by measuring the progress in time of the CdS-Au-driven MB reduction reaction. The results of MB reduction for hybrid CdS-Au NPs were also compared with the properties exhibited by both as-synthesized CdS NPs and free Au nanostructures, as well as by analysing the impact of the amount of HAuCl_4_ used during the CdS-Au NPs synthesis. For the photocatalytic activity measurements, we used CdS-5Au and CdS-7.5Au NPs, as for those systems, the quenching of the luminescence was high enough to ensure a strong interaction between semiconductor and metal parts of the system, thus making the comparison accurate and reliable. In a typical experiment, 1 mL of CdS-Au NPs solution was mixed with 1 mL of distilled water and 1 mL of ethanol. During stirring, 1 mL of MB aqueous solution was added, and further stirred for 10 min to establish an adsorption–desorption equilibrium. A probe of the mixture was taken and its absorption spectrum was measured as the “0” time point using a JASCO V670 spectrophotometer. The mixture was continuously stirred and exposed to light from a solar simulator (OPTEL Fiber illuminator, Opole, Poland). Next, samples for absorbance measurements were taken without filtration at 5, 10, 15, 20, 25, 30, 45, and 60 min after the “0” time point. For comparison, we performed the measurements for CdS-7.5 Au NPs, CdS-5Au NPs, as-synthesized CdS NPs, free Au nanostructures, mixture of CdS NPs and Au nanostructures, and MB itself, keeping the same concentrations of the components. In the case of free Au nanostructures, we added an adequate quantity of Rac-penicillamine solution to keep the same reduction reaction conditions.

#### 2.2.3. Reactive Oxygen Species Generation Measurements

Further, we investigated ROS generation ability of the studied CdS-Au hybrid nanosystems. First, 4 mg of BSA was fully dissolved in 3.5 mL of distilled water. During stirring, 500 μL of CdS-Au NPs aqueous solution was added, and immediately, a sample of the mixture was taken and its luminescence spectrum was measured as the “0” time point using a FluoroMax-4 spectrofluorometer in the wavelength range of 300–425 nm (excitation wavelength: λ = 290 nm). The mixture was continuously stirred and exposed to solar simulator light (OPTEL Fiber illuminator, Opole, Poland), providing 100 mW/cm^2^ with a 320 nm long-pass optical filter in order to preferentially excite the CdS-Au hybrid nanosystems and to avoid the direct photo-degradation of BSA by deep UV light. Next, spectra were measured at 2, 4, 6, 8, and 10 min after the “0” time point. For comparison, we performed the measurements for CdS-2.5Au and CdS-5Au.

#### 2.2.4. Two-Photon Absorption Cross-Section Measurements

Two-photon absorption cross-section measurements of as-prepared CdS-5Au NPs were performed using a laser system that consisted of a Quantronix Integra-C Ti:sapphire regenerative amplifier, producing ~130 fs, 800 nm pulses, with 1 mJ energy per pulse and a 1 kHz repetition rate, and a Quantronix Palitra-FS optical parametric amplifier for wavelength tuning (we tuned the excitation wavelength between 700 nm and 750 nm). Following the calculation procedure described by Makarov et al. [31], we used a fluorescein solution as a reference and an OceanOptics FLAME-T-VIS/NIR fiber spectrophotometer for acquiring the two-photon excited emission spectra.

## 3. Results and Discussion

**Figure 1:**
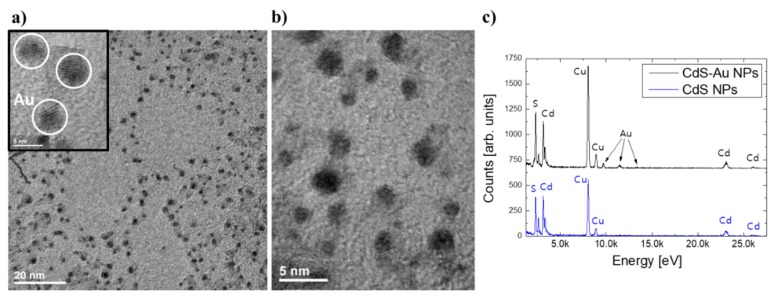
Representative transmission electron microscopy (TEM) and HRTEM images (**a**,**b**) of CdS-5Au nanoparticles (NPs), together with EDX spectrum (**c**) obtained for as-synthesized CdS NPs and CdS-Au hybrid structures. Au nanostructures are visible as the darkest regions (marked in the inset in a with white circles), while CdS tetrapod-shape NPs are more pale owing to the significantly higher density of Au in comparison with CdS.

The morphology of the obtained CdS-Au NPs was investigated based on TEM imaging (Figure 1). As a result of the synthesis, branched CdS NPs were obtained, showing sizes around 20 nm (Figure 1a,b), with Au nanostructures deposited at the surface (inset in Figure 1a shows marked regions of Au). The efficient reduction of HAuCl_4_ was possible only at the surface of the semiconductor, as no additional reducing agent was used during the synthesis process. The synthesis of semiconducting–metal nano-architectures based on nucleation and growth of metallic domains via deposition process at the surface of semiconductor parts is in fact a common strategy to obtain similar hybrid systems [32,33,34,35,36]. The presence of Au domains at the CdS NPs surface was observed as darker spots at TEM images (Figure 1a,b), and additionally confirmed by the EDX measurements (Figure 1c). The as-synthesized raw CdS NPs showed peaks characteristic for cadmium and sulphur elements in the EDX spectra, with additional copper lines arising from the TEM grids. After the second step of the synthesis of CdS-Au NPs, peaks characteristic for gold appeared in the EDX spectra (Figure 1c).

**Figure 2:**
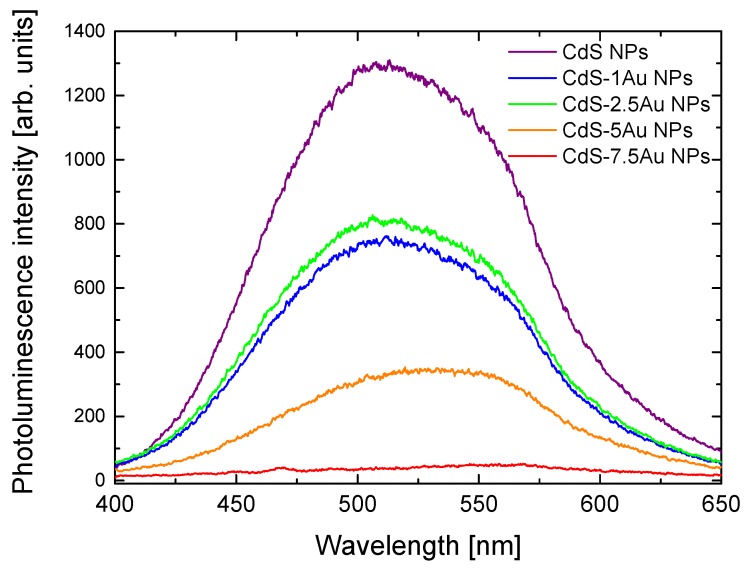
Photoluminescence spectra of CdS NPs showing luminescence quenching in presence of gold domains on CdS NPs’ surface.

The deposition of Au at the surface of CdS NPs strongly influenced their optical properties. Figure 2 presents photoluminescence spectra of as-synthesised raw CdS NPs and hybrid CdS-Au nanostructures, while the corresponding absorbance spectra are shown in Figure S1. A band gap of E_g_ ≈ 2.65 eV was derived from the solution by extrapolating the linear portion of the curve in Figure S1 to zero absorption. The modification of CdS NPs with Au NPs slightly reduces the E_g_ values, proving the photocatalytic activity [37]. The maximum of CdS NPs emission appears at 508 nm. In the case of CdS-Au NPs, the luminescence intensity decreases along with the growth of Au at the CdS surface, confirming direct contact between metal and semiconductor visible in the TEM images, as well as indicating interactions between them. The photoluminescence quenching may be caused by electron transfer from CdS NPs conduction band to Au nanostructures, thus preventing efficient electron–hole recombination and the following radiative band-gap emission. The more gold precursor used in the synthesis, the less intensive the photoluminescence of the CdS-Au nanosystems, until it is completely quenched for CdS-7.5Au, which most probably results from the dependence suggested by Dana et al. [19] that larger metal nanostructures provide more efficient electron transfer from semiconductor nanoparticles to an acceptor.

**Figure 3:**
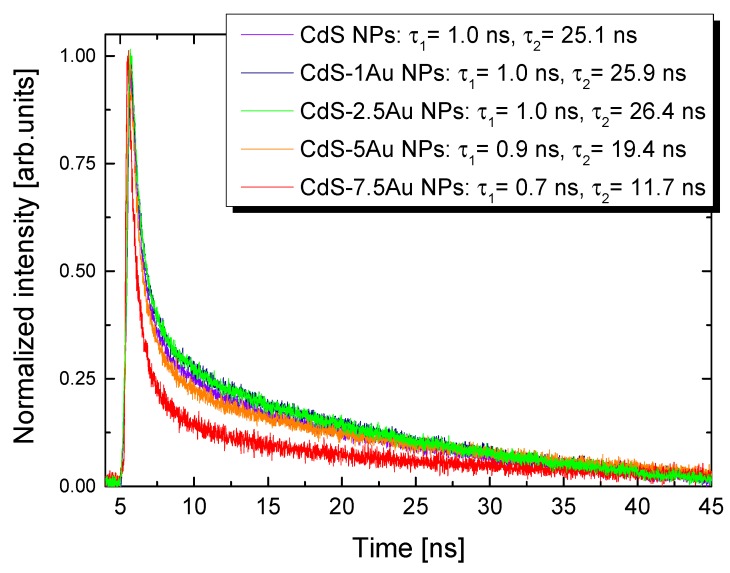
Time-resolved photoluminescence of CdS NPs and CdS-Au nanostructures together with the calculated luminescence lifetimes.

Juxtaposition of the time-resolved photoluminescence decay curves (Figure 3) shows that the presence of Au nanostructures at the CdS NPs surface reduces the lifetimes of CdS NPs, especially for CdS-5Au and CdS-7.5Au samples. The calculated τ values (short and long components) for as-synthesized CdS NPs were close to those reported by us for similar systems [23] (full exponential fitting parameters can be found in Table S1), and decreased with increasing Au concentrations; the strongest lifetime reduction (approximately 50%) was observed for CdS-7.5Au NPs. Those results are consistent with the photoluminescence quenching studies, suggesting that the electron transfer occurs faster in heteroparticles with larger metal domains. A possible explanation is that there are more electron-accepting sites in larger Au nanostructures, enhancing their ability to trap electrons [19].

**Figure 4:**
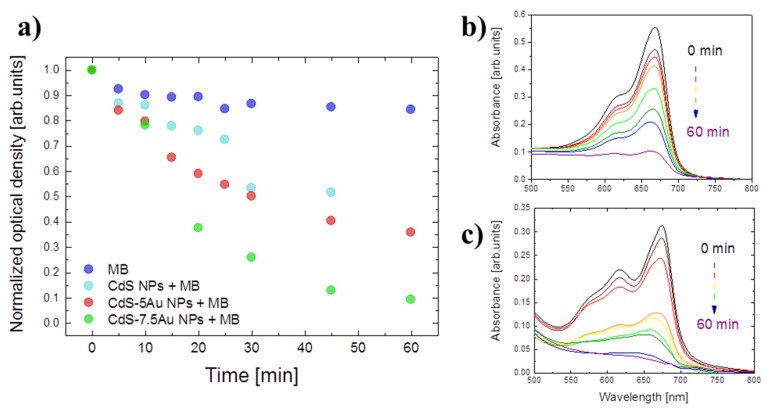
Optical density maxima changes at ~668 nm of methylene blue (MB) under prolonged irradiation: the dye itself (blue) and in the presence of CdS (light blue), CdS-5Au NPs (red), or CdS-7.5Au NPs (green) (**a**). Absorbance spectra changes in time for MB irradiated in the presence of CdS-5Au NPs (**b**) and CdS-7.5Au NPs (**c**).

Two types of experiments were performed in order to show how the charge separation at the CdS-Au interface can be used for certain applications. CdS-Au hybrid nanosystems’ ability to catalyse sunlight-driven reduction reactions was performed at ambient temperature in water environment, and the results of MB absorbance bleaching are presented in Figure 4. Light exposure influences MB itself only at the beginning, and then its absorption spectrum is practically stable in time, even after an hour of the continuous irradiation (blue dots in Figure 4). However, when mixed with CdS-Au NPs and exposed to light, the MB absorption decreases with time; this behavior is observed for both CdS-5Au NPs and CdS-7.5Au NPs samples (red and green dots at Figure 4, respectively). This phenomenon results from separation of positively charged holes trapped by hole scavenger from negatively charged electrons accepted on metal, which are then available for reactions; in this case, for MB reduction, which losses its absorption ability in its reduced form. A significant difference between the photocatalytic ability of CdS-5Au and CdS-7.5Au is worth attention, as after an hour of light exposure, 64% and 91% of MB was degraded, respectively, confirming faster electron transfer to large metal domains suggested by photoluminescence and time-resolved photoluminescence studies. Absorption measurements of MB in the presence of only CdS NPs showed that they possess little photocatalytic activity, definitely much smaller than the hybrid nanosystems. We also performed a series of reference experiments, including MB in presence of free Au NPs (Figure S2 shows absorbance spectra of obtained NPs) in two concentrations: 5 mM and 7.5 mM (called 5Au NPs and 7.5Au NPs respectively), as well as physical mixtures of free Au NPs in both concentrations with as-synthesized CdS NPs, and the results are shown in Figures S3–S5. The best results were obtained for CdS-7.5Au hybrid NPs, showing significantly more efficient degradation of MB upon light exposure than for the physical mixture of CdS NPs and 7.5Au NPs (Figure S4) or samples with lower Au content (Figure S4).

**Figure 5:**
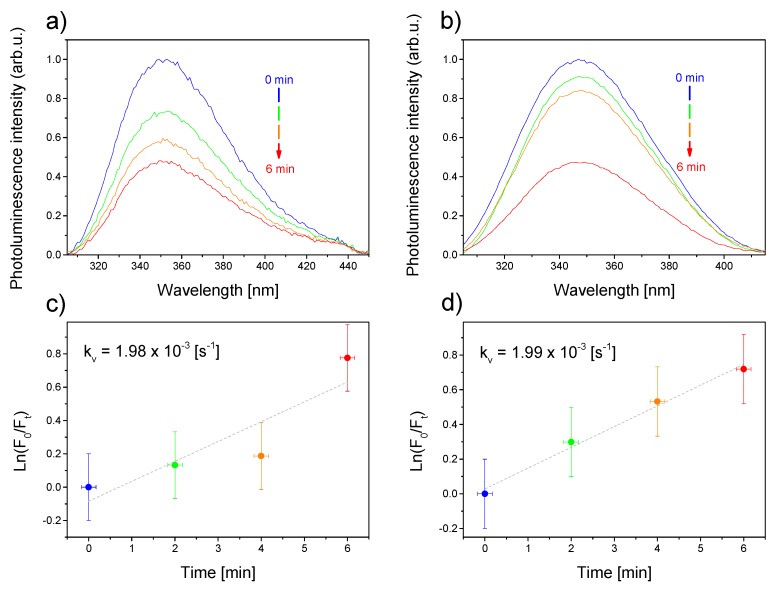
Photoluminescence quenching of bovine serum albumin (BSA) in time in the presence of CdS-2.5Au NPs (**a**) and CdS-5Au NPs (**b**). Kinetic curves for the photooxidation of BSA in the presence of CdS-2.5Au NPs (**c**) and CdS-5Au NPs (**d**).

As shown in previous experiments, the presence of Au deposited at the CdS NPs’ surface can effectively prevent the electron–hole recombination. The electron transferred to the metal domain could then be used for light-driven catalytic reactions. In the second experiment, we also investigated the possibility of as-synthesized nanosystems to generate ROS, as a result of light-induced electron–hole generation, followed by the effective charge separation owing to the heterogeneous character of the studied system. The measurements were performed based on detecting ROS-sensitive photoluminescence of BSA decrease, when mixed with CdS-Au NPs and exposed to broad-band light. Figure 5 shows photoluminescence decrease of BSA in the presence of CdS-2.5Au NPs (Figure 5a) and CdS-5Au NPs (Figure 5b), showing a maximum at 347 nm upon excitation at 290 nm. Regardless of the concentration of Au, the integrated photoluminescence intensity decreased by slightly more than 50% for CdS-2.5Au NPs and CdS-5Au NPs, with a faster response observed for the system with a higher Au concentration, showing additionally the proof-of-concept of the CdS-Au NPs’ ability to generate ROS upon light exposure. For better comparison, on the basis of the slopes of the semi-log plots, the first-order rate constants (k_v_) of the observed photo-process were further calculated [38,39] for systems with CdS-2.5Au NPs (Figure 5c) and CdS-5Au NPs (Figure 5d). The pseudo first-order rate constants k_v_ of this photo-process were approximately 1.98 × 10^−3^ and 1.99 × 10^−3^ s^−1^ for CdS-2.5Au NPs and CdS-5Au NPs, respectively. Those values are one order of magnitude higher than the corresponding ones measured and calculated by us for ZnO NPs [38], which establishes the hybrid materials studied here also as potential efficient ROS generating anti-microbial and anti-cancer agents in photodynamic-based treatments. The main ROS types produced in the presence of very similar systems [40] are hydroxyl radicals, resulting from water oxidation reaction using separated holes, as well as hydrogen peroxide and superoxides, which are products of oxygen reduction. However, when measuring BSA photoluminescence in the presence of hole scavenger (i.e., preventing water oxidation), we observed hardly a detectable signal, which did not display exposure time–intensity correlation. On the other hand, when no hole scavenger was added, enabling water oxidation reaction to occur, we observed clear BSA photoluminescence signal (Figure 5a,b), whose intensity decreased exponentially (Figure 5c,d). This result suggests that the oxygen reduction reaction is hard to control as the amount of oxygen in water varies, but also that this reaction contributes significantly less to the total ROS production than the water oxidation reaction, owing to the low concentration of oxygen in water (averagely about 10 $\frac{mg\;O_{2}}{L}$). Therefore, we suppose that the main ROS type produced in presence of CdS-Au is hydroxyl radicals.

**Figure 6:**
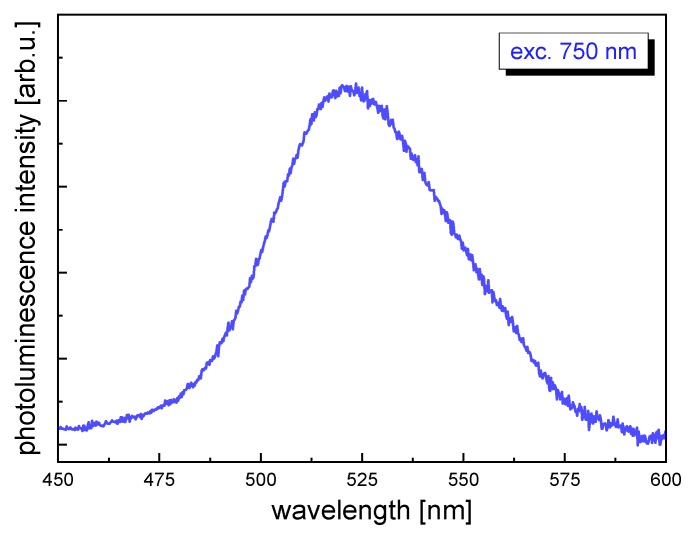
Representative photoluminescence spectra of CdS-5Au NPs upon two-photon 750 nm excitation.

On the basis of the results presented above, we selected the CdS-5Au NPs sample for NLO measurements as it exhibited excellent reduction (Figure 4a,b) and oxidation (Figure 5b) properties, and it simultaneously displayed photoluminescence intensity that was high enough (Figure 2) to be detectable and comparable in the TPEE technique. Figure 6 shows photoluminescence spectra of CdS-5Au upon femtosecond laser excitation at 750 nm, with the maximum at approximately 520 nm.

Two-photon absorption cross-section calculation results obtained in this work are presented in Table 1 and compared to the values already reported for identically synthesized CdS NPs [23]. The CdS-5Au NPs absorption cross section reaches 15.8 × 10^3^ GM upon excitation at 725 nm, exceeding the corresponding value for CdS NPs. Scaling those values with the molecular weight (MW) of single particle, the values of σ_2_/MW obtained for CdS-5Au NPs are also higher than the corresponding ones obtained for CdS NPs. We attribute this increase to the combination of semiconductor’s exciton band edge absorption and plasmon resonance resulting from the presence of Au NPs [41]. For applications based on two-photon brightness, it is essential to compare not solely the two-photon absorption cross sections, but rather the two-photon brightness, because significant photoluminescence quenching caused by electron transfer from the CdS NPs conduction band to Au nanostructures might have a high impact on two-photon excited emission, even if two-photon absorption cross sections suggest a strong nonlinear optical response. Therefore, Table 1 also presents the σ_2_×QY parameter considering quantum yield values. The resulting two-photon brightness of CdS-5Au NPs is comparable (even slightly higher) to previously reported values for CdS NPs, suggesting that the resonance effect counteracts the quenching effect, maintaining two-photon brightness at a high level. Interestingly, embedment of gold nanostructures on CdS NPs not only significantly enhances their reduction and oxidation capabilities, making them promising candidates for photocatalytic and photosensitizing applications, but also preserves their two-photon brightness so that the resulting material may perform multiple functions at the same time.

## 4. Conclusions

On the basis of the results presented above, we conclude that we successfully synthesized hybrid CdS-Au NPs, which exhibit triple photocatalytic, photosensitizing, and nonlinear optical properties. The important advantage of the proposed colloidal systems stems from the water-based, low temperature synthesis process, which allows for straightforward (no additional surface treatment is needed) application of the obtained systems, especially in sun-light driven photocatalysis or broad-band light sources, triggered photodynamic therapy (see Figure S6 for the proposed scheme of photodegration mechanisms). TEM images of CdS-Au nanohybrids and decrease of photoluminescence intensity, as well as lifetime shortening in comparison with as-synthesised raw CdS NPs, allow us to state that Au was effectively deposited at the surface of semiconductor NPs. The charge separation phenomenon was observed in the presence of CdS-Au NPs as a function of time, based on MB absorbance bleaching measurements study and upon light exposure and used for proof-of-concept specific application. Absorbance of MB decreased when exposed to light in the presence of CdS-Au NPs, suggesting that electrons were generated on CdS NPs, separated by transfer to Au domain, and they reinforced MB reduction reaction showing the photocatalytic efficiency of the obtained systems. Moreover, we report higher photocatalytic activity in the case of higher Au concentration used during the synthesis process of CdSe-Au NPs. BSA photoluminescence intensity decreased in time with a k_v_ rate constant of 1.99 × 10^−3^ s^−1^ in the presence of CdS-5Au NPs exposed to light, suggesting efficient ROS generation. This feature shows the potential of CdS-Au NPs as anti-microbial or anti-cancer agents in light triggered reactions. Despite the reduction of quantum yield caused by the addition of metal domains, which prevent excitons recombination, we selected an optimal concentration of Au nanostructures in order to simultaneously achieve photocatalytic and photosensitizing properties without forfeiting the two-photon absorption ability, which we report to reach 15.80 × 10^3^ GM and two-photon brightness (1.58 × 10^3^ GM). This unusual combination in one multifunctional material may find future application as a novel type of theranostic that would unite cancer cells two-photon imaging with their necrosis caused by ROS generation in oxidation reaction or bioimaging with inflammation treatment by free radicals neutralization in reduction reaction, depending on the conditions.

## Figures and Tables

**Table 1:** Two-photon absorption cross sections σ_2_ and two-photon brightness σ_2_ × QY of as-prepared CdS-Au NPs in comparison with CdS NPs characterized in a previous work [23]. Exc. λ—excitation wavelength. QY, quantum yield.

Sample	exc. λ (nm)	σ_2_ (GM)	σ_2_ × QY (GM)	σ_2_/MW (GM)	Ref.
CdS	700	6 × 10^2^	3.66 × 10^2^	1× 10^−3^	
	725	5.7 × 10^2^	3.42 × 10^2^	0.6 × 10^−3^	[23]
	750	2.3 × 10^2^	1.38 × 10^2^	0.2 × 10^−3^	
CdS-5Au	700	7.0 × 10^3^	7.0 × 10^2^	9 × 10^−3^	this work
	725	15.8 × 10^3^	1.58 × 10^3^	20 × 10^−3^	
	750	6.9 × 10^3^	6.90 × 10^2^	9 × 10^−3^	

## Supplementary Materials

The following are available online at https://www.mdpi.com/2079-4991/10/4/715/s1, Figure S1: Absorbance spectra of CdS NPs and CdS-Au NPs obtained in the presence of different Au concentrations. Figure S2: Absorbance spectra of free Au NPs prepared using different concentrations of HAuCl_3_. Figure S3: Absorbance maxima changes at 668 nm of MB exposed to light: the dye itself (blue) and in the presence of 5Au NPs (pink), 7.5Au NPs (yellow), CdS NPs (light blue), CdS-5Au NPs (red), CdS NPs mixed with 5Au NPs (orange), CdS-7.5Au NPs (light green), and CdS NPs mixed with 7.5Au NPs (dark green) in time. Figure S4: Absorbance maxima changes at 668 nm of MB exposed to light in the presence of 5Au NPs (yellow), CdS-5Au NPs (green), and CdS NPs mixed with 5Au NPs (red) in time. Figure S5: Absorbance maxima changes at 668 nm of MB exposed to light in the presence of 7Au NPs (yellow), CdS-7.5Au NPs (green), and CdS NPs mixed with 7.5Au NPs (red) in time. Figure S6: Schematic representation of possible photodegration mechanisms observed in the investigated CdS-Au hybrid nanostructures. Table S1: Luminescence lifetimes fitting parameters obtained for CdS NPs and CdS-Au nanostructures.
