# Supplementary material for: Functional CdS-Au Nanocomposite for Efficient Photocatalytic, Photosensitizing, and Two-Photon Applications
Source: Nanomaterials (Basel). 2020 Apr 10;10(4):715. doi: 10.3390/nano10040715 (PMC7221832; doi:10.3390/nano10040715)
Supplement: Supplementary file 1 [file nanomaterials-10-00715-s001.pdf]

Supplementary Information

Functional CdS-Au Nanocomposite for Efficient Photocatalytic, Photosensitizing, and Two-Photon Applications

Katarzyna C. Nawrot [1], Dominika Wawrzyńczyk [1], Oleksii Bezkrovnyi [2], Leszek Kępiński [2], Bartłomiej Cichy [2], Marek Samoć [1], and Marcin Nyk [1,*]

[1] Advanced Materials Engineering and Modelling Group, Faculty of Chemistry, Wroclaw University of Science and Technology, Wybrzeze Wyspianskiego 27, 50-370 Wroclaw, Poland; katarzyna.nawrot@pwr.edu.pl (K.C.N.); dominika.wawrzynczyk@pwr.edu.pl (D.W.); marek.samoc@pwr.edu.pl (M.S.)

[2] W. Trzebiatowski Institute of Low Temperature and Structure Research Polish Academy of Sciences, Okólna 2, 50-422 Wroclaw, Poland; o.bezkrovnyi@int.pan.wroc.pl (O.B.); l.kepinski@int.pan.wroc.pl (L.K.); b.cichy@int.pan.wroc.pl (B.C.)

* Correspondence: marcin.nyk@pwr.edu.pl; Tel.: +48-71-320-2316

Received: 18 March 2020; Accepted: 7 April 2020; Published: date

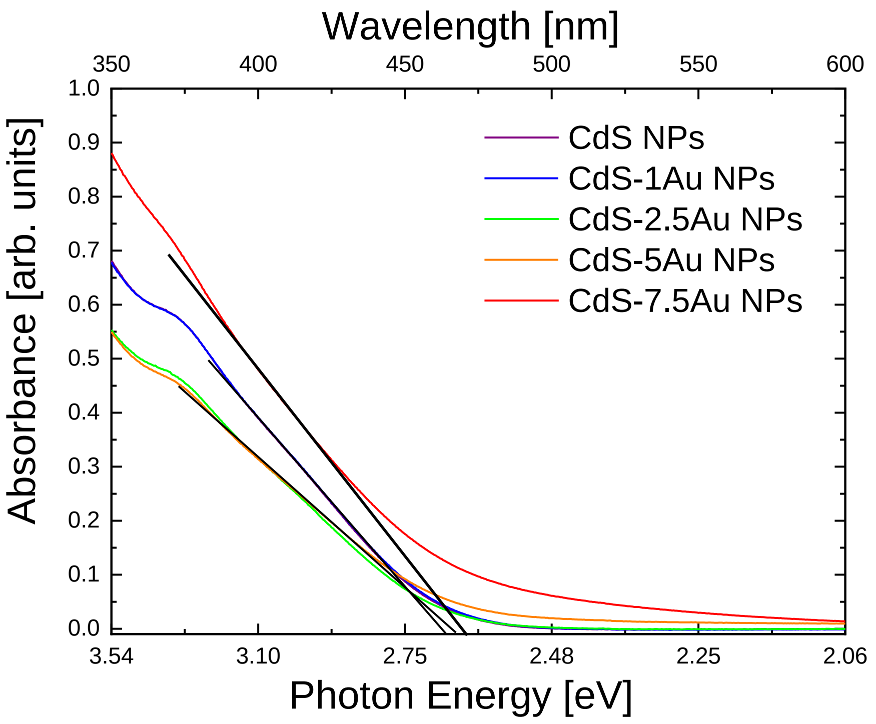

Figure S1. Absorbance spectra and Tauc plots of CdS NPs and CdS-Au NPs obtained in the presence of different Au concentrations.

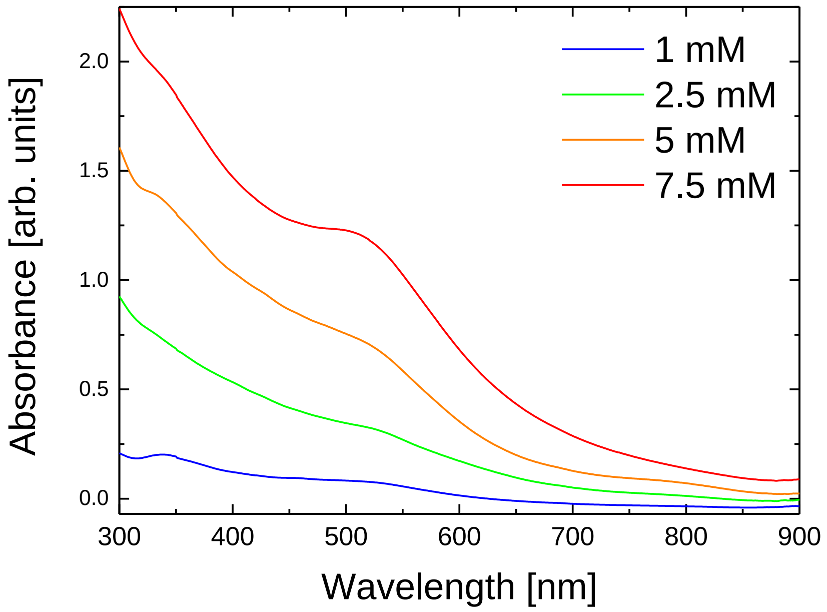

Figure S2. Absorbance spectra of free Au NPs prepared using different concentrations of $HAuCl_3$.

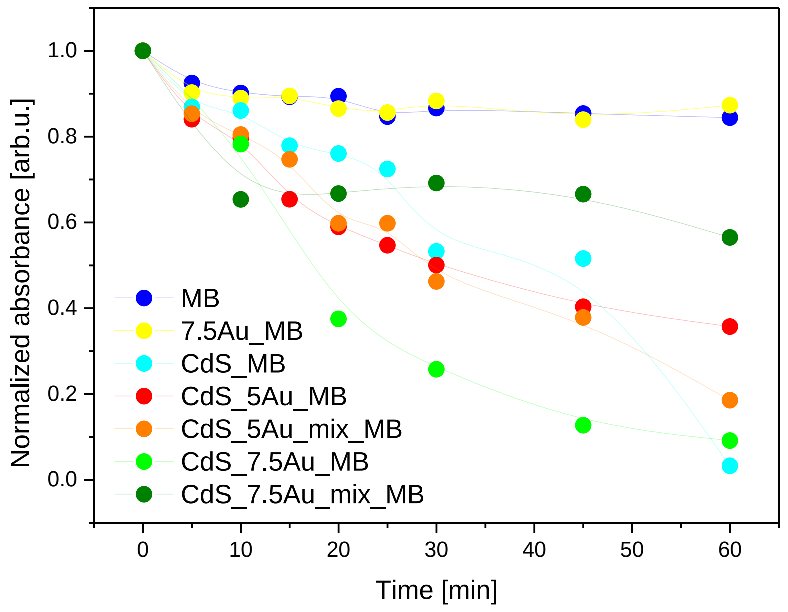

Figure S3. Absorbance maxima changes at 668 nm of MB exposed to light: the dye itself (blue) and in the presence of 5Au NPs (pink), 7.5Au NPs (yellow), CdS NPs (light blue), CdS-5Au NPs (red), CdS NPs mixed with 5Au NPs (orange), CdS-7.5Au NPs (light green) and CdS NPs mixed with 7.5Au NPs (dark green) in time. Lines are added to guide eyes.

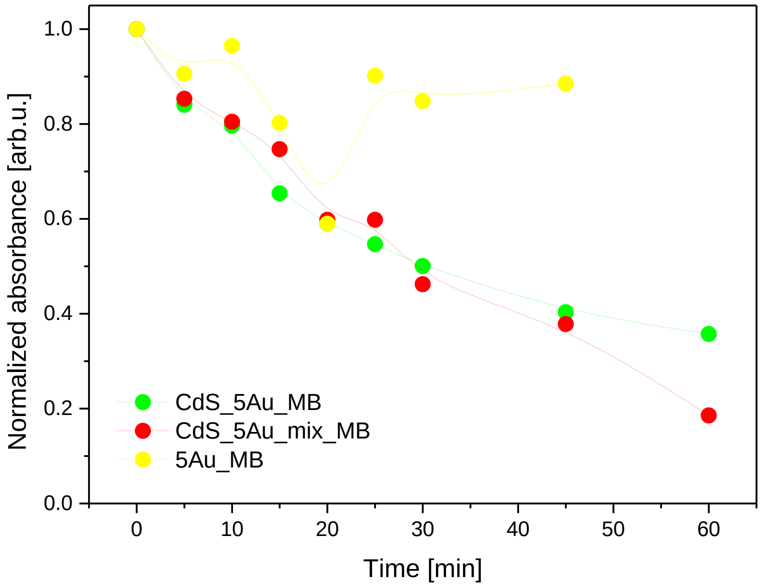

Figure S4. Absorbance maxima changes at 668 nm of MB exposed to light in the presence of 5Au NPs (yellow), CdS-5Au NPs (green) and CdS NPs mixed with 5Au NPs (red) in time. Lines are added to guide eyes.

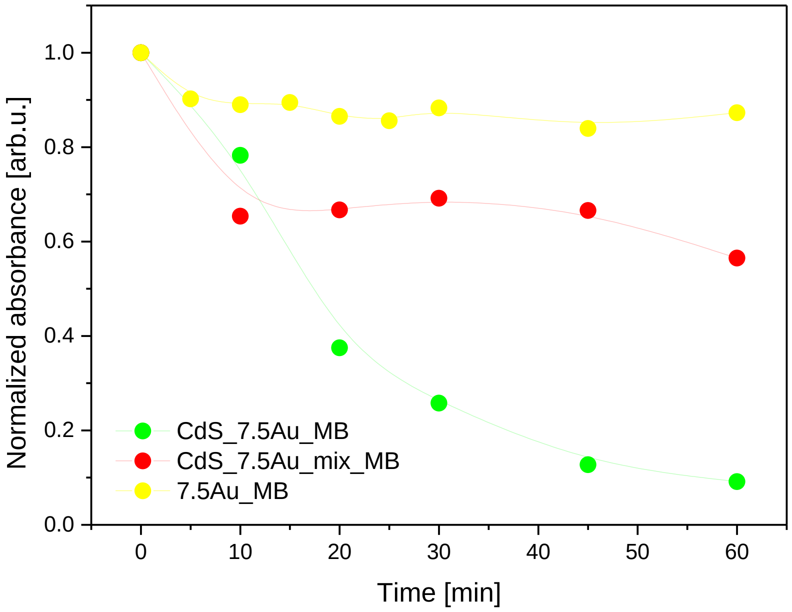

Figure S5. Absorbance maxima changes at 668 nm of MB exposed to light in the presence of 7Au NPs (yellow), CdS-7.5Au NPs (green) and CdS NPs mixed with 7.5Au NPs (red) in time. Lines are added to guide eyes.

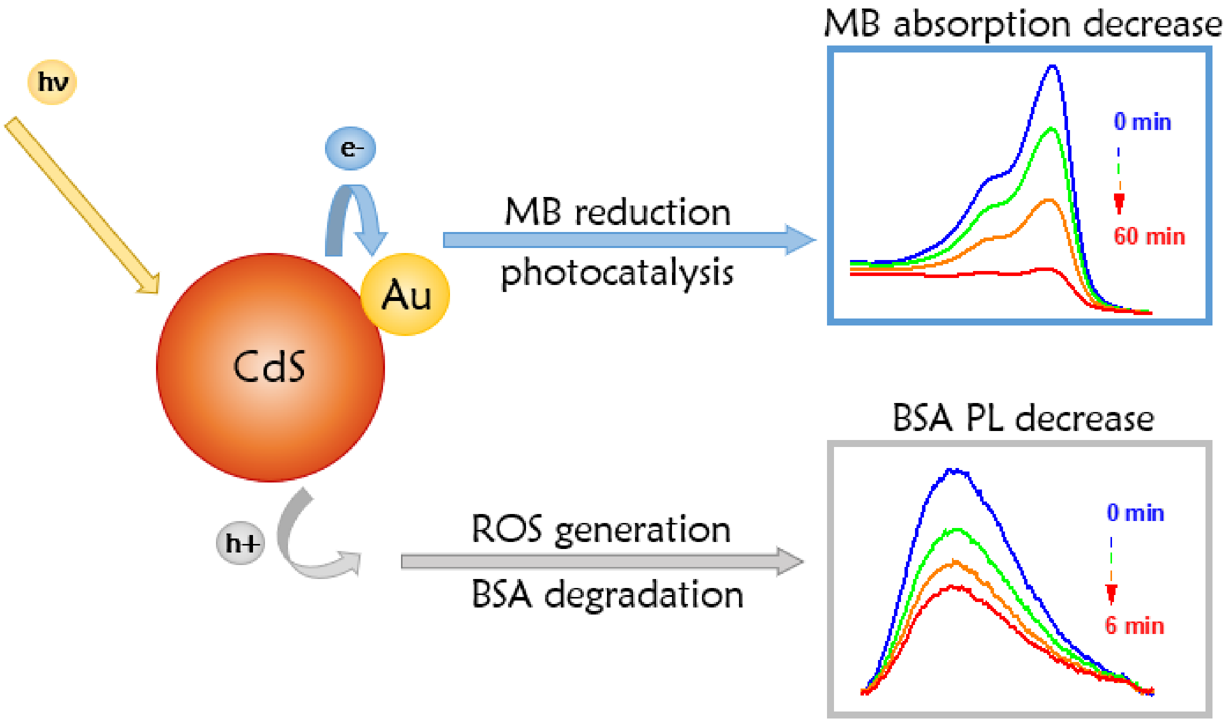

Figure S6. Schematic representation of possible photodegration mechanisms observed in the investigated CdS-Au hybrid nanostructures.

Table S1. Luminescence lifetimes fitting parameters obtained for CdS NPs and CdS-Au nanostructures.

**Sample**	**A_1 [10^6]**	**τ_1 [ns]**	**A_2 [10^3]**	**τ_2 [ns]**
CdS NPs	2.4	1.0	6.0	25.1
CdS-1Au NPs	2.7	1.0	6.6	25.9
CdS-2.5Au NPs	1.6	1.0	5.5	26.4
CdS-5Au NPs	1.3	0.9	1.8	19.4
CdS-7.5Au NPs	1.7	0.7	0.4	11.7
